# Intergenogroup Recombinant Sapovirus in Japan, 2007–2008

**DOI:** 10.3201/eid1507.090153

**Published:** 2009-07

**Authors:** Wisoot Chanit, Aksara Thongprachum, Pattara Khamrin, Shoko Okitsu, Masashi Mizuguchi, Hiroshi Ushijima

**Affiliations:** The University of Tokyo, Tokyo, Japan (W. Chanit, A. Thongprachum, M. Mizuguchi, H. Ushijima); Aino University, Tokyo (P. Khamrin, H. Ushijima); Aino College, Tokyo (S. Okitsu)

**Keywords:** Sapovirus, viruses, recombination, gastroenteritis, enteric diseases, Japan, dispatch

## Abstract

We investigated the incidence of sapovirus (SaV)–associated gastroenteritis in infants and children in Japan during 2007–2008 and characterized the diversity of SaV-positive strains. SaV was detected in 19 (4%) of 477 fecal specimens. The leading genogroup (79%, 15 cases) comprised intergenogroup recombinant SaVs (GII/GIV).

Sapovirus (SaV) is now considered a notable global enteropathogen of acute gastroenteritis in persons of all ages (*1**–**3*). As a member of the family *Caliciviridae*, SaV has a single-stranded positive-sense RNA genome of ≈7.3–7.5 kb that contains either 2 or 3 main open reading frames (ORFs 1–3). SaV ORF1 encodes for nonstructural proteins and the major capsid protein (VP1), and ORF2 (VP2) and ORF3 encode proteins of yet unknown functions. On the basis of VP1 nucleotide sequences, SaVs are divided into 5 genogroups (GI–GV), of which GI, GII, GIV, and GV strains are known to infect humans; SaV GIII infects porcine species. We investigated the incidence of SaV-associated gastroenteritis in infants and children in Japan during 2007–2008 and characterized the diversity of SaV-positive strains.

## The Study

We collected 477 fecal specimens from nonhospitalized children with acute gastroenteritis in pediatric clinics in 5 localities in Japan (Tokyo, Sapporo, Saga, Osaka, and Maizuru) during July 2007–June 2008. Of these, 14 specimens were from Tokyo, 30 from Sapporo, 77 from Saga, 91 from Osaka, and 265 from Maizuru. We defined diarrhea as at least 3 passages of unformed (loose and watery) feces a day. We defined acute gastroenteritis as diarrhea and other symptoms, such as vomiting, fever, and abdominal pain. Children studied ranged in age from 1 month to 14 years (median 25 months). The Ethical Committees of The University of Tokyo and Aino University approved the study. A parent or guardian of each child provided informed consent.

RNA was extracted and purified by using the QIAamp Viral RNA Mini kit (QIAGEN, Hilden, Germany), according to the manufacturer’s instructions. Reverse transcription (RT) was performed with 5 µL of RNA template by using SuperScript III reverse transcriptase (Invitrogen, Carlsbad, CA, USA). By using multiplex RT-PCR, 2 groups of diarrheal viruses were identified: 1) astrovirus, norovirus, and sapovirus and 2) rotavirus and adenovirus (*2*). The PCR products were analyzed by 1.5% agarose gel electrophoresis and visualized by staining with SYBR Safe (Invitrogen). Nucleotide sequences were determined by using a Big-Dye terminator cycle sequencing kit and ABI Prism 310 Genetic Analyzer (Applied Biosystems, Foster City, CA, USA). The nucleotide sequences were aligned by using ClustalX (http://bips.u-strasbg.fr/fr/Documentation/ClustalX), and distances were calculated by using the 2-parameter Kimura method. Phylogenetic trees with bootstrap analysis from 1,000 replicas were generated by using the neighbor-joining method. The sequences of SaV strains detected in the study have been submitted to GenBank under accession nos. FJ445092–FJ445110.

Of the viruses isolated from diarrhea samples, group A rotavirus was the most prevalent (20.5%), followed by norovirus (19.3%), adenovirus (4.4%,) sapovirus (3.9%), group C rotavirus (0.8%), and astrovirus (0.2%). In addition, we found viral mixed infections in 1.8%.

Sapovirus was detected in 19 (4%) fecal specimens. The highest incidence of SaV infection was in the 1-year-old group (9 [47%]), and most (13 [68%]) of these infections occurred in infants and children <3 years of age. Infections increased slightly during December through February (12 cases). The most common signs and symptoms in SaV-infected children were diarrhea (19 children [100%]), fever >100°F (5 [26%]), and vomiting >3 times a day (3 [15%]).

**Figure 1:**
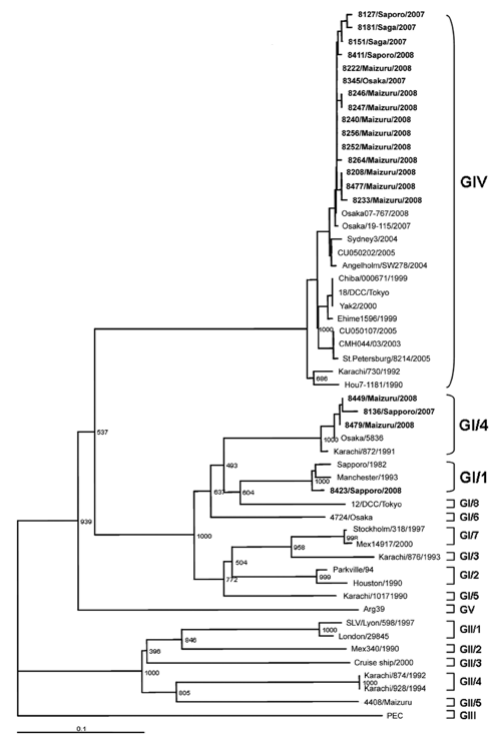
Phylogenetic tree of nucleotide sequences of sapovirus (SaV) strains (shown in **boldface**). The tree was constructed from partial nucleotide sequences of the capsid region by using PEC strain (a porcine SaV) as an outgroup. The numbers on each branch indicate the bootstrap values. Scale bar indicates nucleotide substitutions per position. GenBank accession numbers of reference strains are as follows: Osaka07-767/08/JP (AB433785), Osaka/19-115/07/JP (AB327280), Sydney3/04/AU (DQ104357), CU050202/05/HK (DQ155647), Angelholm/SW278/04/SE (DQ125333), Chiba/000671/99/JP (AJ786349), 18/DCC/Tokyo/43/JP (AB236378), Yak2/00/JP (AB046353), Ehime1596/99/JP (AM049952), CU050107/05/HK (DQ155646), CMH044/03/03/THA (EF600796), St.Pertersburg/8214/05/RUS (FJ214057), Karachi/730/92/PK (AB126249), Hou7-1181/90/USA (AF435814), Osaka/5836/04/JP(AB242324), Karachi/872/91/PK (AB181231), Sapporo/82/JP (U65427), Manchester/93/UK (X86560), 12/DCC/Tokyo/44/JP (AB235380), 4724/Osaka/02/JP (AB180212), Stockholm/318/97/SE (AF194182), Mex14917/00/USA (AF435813), Karachi/867/93/PK (AB181132), Parkville/94/UK (U73124), Houston/90/USA (U95644), Karachi/1017/90/PK (AB181227), Arg39/95/ARG (AY289803), Lyon/598/97/F (AJ271056), London/29845/92/UK (U95645), Mex340/90/USA (AF435812), Cruise ship/00/USA (AY289804), Karachi/874/92/PK (AB181129), Karachi/928/94/PK (AB181128), 4408/Maizuru/03/JP (AB180209), and PEC (AF182760).

Nineteen SaV sequences were analyzed by phylogenetic analysis and grouped by using the recent SaV capsid region classification scheme (*4*). Most SaV sequences belonged to genogroup IV (15 cases [79%]), followed by GI/4 (3 cases [16%]), and GI/1 (1 case [5%]). Three sequences of GI/4 genotype had 98%–100% nucleotide identity with each other and grouped with the Karachi/872/91/PK and Osaka/5836/JP strains known to belong to GI/4 genotype. One GI/1 sequence had 97% nucleotide identity and clustered with Manchester sequence (Figure 1).

**Figure 2:**
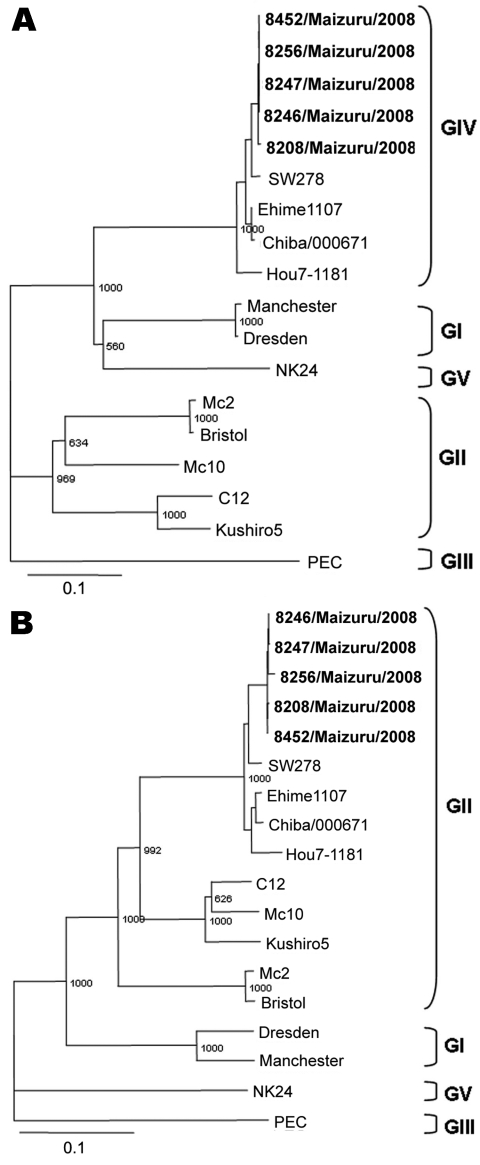
Phylogenetic analysis of the polymerase region (A) and the capsid region (B), showing the different genogroups (GI–GV). The sapovirus (SaV) isolates detected in the study are highlighted in **boldface**. The scale indicates nucleotide substitutions per position. The numbers in the branches indicate the bootstrap values. GenBank accession numbers of reference strains are as follows: C12 (AY603425), Mc10 (AY237420), Kushiro5 (AB455793), Mc2 (AY237419), Bristol (AJ249939), Dresden (AY694184), NK24 (AY646856), Ehime1107 (DQ058829), Yak2 (AB046353), and Yokohama/16/2007/JP (AB305049).

Nucleotide sequence comparison of the identified 15 GIV shared little or almost no divergence among themselves (98%–100% identity), even when they were detected in regions of Japan distant from each other. They are likely to represent a single strain, 8208/Maizuru/08/JP. The 8208/Maizuru/08/JP sequence closely matched Ehime1107 and SW278 (*5*), and Yak2 (*6*) sequences, which were previously established as intergenogroup recombinant SaV strains with the GII polymerase region and GIV capsid region, with 97% and 96% nucleotide identities, respectively. To determine whether our GIV strains were the recombinant SaV, 5 of the 15 GIV strains were randomly selected as representative, and long genomic fragments that included part of the RNA polymerase and part of the capsid genes were amplified by using primers SR80/2 (5′-TGGGATTCTACACAAAACCC-3′) and SLV5749 (5′-CGGRCYTCAAA VSTACCBCCCCA-3′), which generated a 1,151-bp product. The products were directly sequenced, and capsid- and polymerase-based phylogenetic trees confirmed these strains as the recombinant SaVs (Figure 2). We suggest the GIV strains isolated in our study were intergenogroup recombinants. Also, these recombinant strains were detected in 4 locations distant from each other: Maizuru city (10 cases), Sapporo and Saga (2 cases each), and Osaka (1 case), which suggests that the recombinant strains were widely spread through the country.

## Conclusions

According to the past 5 years of SaV surveillance conducted in the same setting and population in Japan, SaV GI/1 was the most common genotype during 2003–2004, and thereafter genotype GI/6 dominated over the GI/1 in 2004–2005 (*2*). Then, the GI/6 genotype was replaced by the predominant SaV GI/1 since 2005 until 2007 (*7*; S.K. Dey, unpub. data). Although GIV SaV strains had been isolated in some countries, including Japan, Thailand, Pakistan, and Hong Kong Special Administrative Region of the People’s Republic of China (*4**,**8**–**11*), they were detected less often than strains from the other genogroups, and whether they are the recombinant strains has not been confirmed. These findings demonstrate the changing epidemiology of SaV genogroup with the emergence of intergenogroup recombinant SaVs (GII/GIV) and the sudden decrease of predominant SaV GI in Japan during 2007–2008.

Recently, several types of recombinant SaV strains, including intergenogroup and intragenogroup, have occurred frequently in Japan (*5**,**12**–**14*), which indicates that recombination between SaV genomes is another important feature of the evolution of SaV. Although factors contributing to the emergence of these recombinant stains during the period of surveillance are not known, the recombinant strains possibly appeared when the pediatric population might have lacked antibody protection to these strains.

Our results suggest that recombinant strains may be underestimated. Characterization of SaVs usually is based on the capsid gene sequence only, whereas both the capsid gene and RNA polymerase sequences are needed to identify such viruses. Furthermore, constant surveillance is important to successfully monitor the emergence of these strains.
